# 717 Survival Benefit with a Topical Anti-inflammatory with Antimicrobial Properties in Patients with >15%; TBSA Burns

**DOI:** 10.1093/jbcr/irae036.261

**Published:** 2024-04-17

**Authors:** Myles H E Dakin, Tom D Kenny, Richard Aspinall

**Affiliations:** Hypo-Stream, Cambridge, England; Hypo-Stream Ltd, Waterlooville, England; Hypo-Stream Ltd, Cambridge, England; Hypo-Stream, Cambridge, England; Hypo-Stream Ltd, Waterlooville, England; Hypo-Stream Ltd, Cambridge, England; Hypo-Stream, Cambridge, England; Hypo-Stream Ltd, Waterlooville, England; Hypo-Stream Ltd, Cambridge, England

## Abstract

**Introduction:**

Burn units acknowledge the challenges of burn-site infection including risk to graft survival and IL-6 mediated systemic inflammatory response, sepsis, concurrent multiple organ failure and death. The risk of repeated grafts, additional ICU time and mortality correlates highly with Pseudomonas spp infection. This risk is compounded by high levels of AMR species (up to 50%) in burns units. The mortality and economic burden are significant and likely to worsen without innovation as AMR becomes increasingly prevalent.

**Methods:**

A novel drug, based on an endogenous immunomodulatory compound (a physiologic aqueous chlorine with the acronym PAC) has been shown to possess broad anti-IL6, anti-microbial and keratinocyte activation activity. In vitro studies confirm efficacy against pathogens including AMR species. The compound is not an antibiotic and is termed compound-PAC.

Over a 50 month period, a tertiary burns unit undertook a proof of concept, open label trial to assess the impact of the compound on survival in patients with confirmed Gram -ve bacterial infections in patient with >15% TBSA. Patients with Gram -ve infection entered the study arm of the trial. Aqueous compound-PAC was administered topically via both hydrotherapy showers and soaked dressings, on a 4-6 hourly basis. In the control arm, showering was performed with normal sterile saline at every dressing change.

Data recorded age, gender, depth of burn + TBSA affected, confirmed Pseudomonas infection, length of stay, and overall survival.

**Results:**

Results from audit analysis (blinded to treatment) revealed compound-PAC could be used to manage confirmed Gram -ve infection of burn-sites; clearing colonization of the wounds without adverse effect and maintaining this with continued use. Such treatment was also effective against other bacterial pathogens. Comparison of survival data of patients with confirmed infection treated with compound-PAC (n = 46) against patients without infection and not receiving this treatment (n= 94), revealed that study arm treatment statistically significantly improved survival compared with normal gold standard of recommended clinical care HR = 0.459 (95% CI 0.282 – 0.750 – P< 0.002). In addition, in patients with >50% TBSA, the use of compound-PAC doubled survival vs. control treatment.

**Conclusions:**

Odds ratio analysis concluded patients receiving the study compound had 5x increased probability of survival vs the current standard of care.

**Applicability of Research to Practice:**

The compound has ability to integrate into standard of care as a wash-drench, with potential survival, wound-healing and health-economic benefits. Further, potential, benefit may be the reduction on failure of skin grafts with reduced requirement for surgeries. A separate investigation is exploring the potential for use in treating inhalation burns.

